# Supplementary material for: Identification of photoperiod-induced specific miRNAs in the adrenal glands of Sunite sheep (Ovis aries)
Source: Front Vet Sci. 2022 Jul 22;9:888207. doi: 10.3389/fvets.2022.888207 (PMC9354845; doi:10.3389/fvets.2022.888207)
Supplement: Supplementary file 1 [file Data_Sheet_1.ZIP › Supplementary file/miRDeep2 file.zip!/miRDeep2 file/Novel_Structure/SPLP42A2/Novel_282.pdf]

Provisional ID	:	NC_019458.2_1927
Score total	:	38.9
Score for star read(s)	:	3.9
Score for read counts	:	31.2
Score for mfe	:	2.2
Score for randfold	:	1.6
Score for cons. seed	:	
Total read count	:	73
Mature read count	:	72
Loop read count	:	0
Star read count	:	1

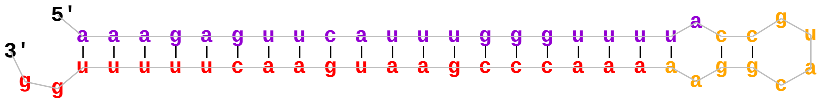

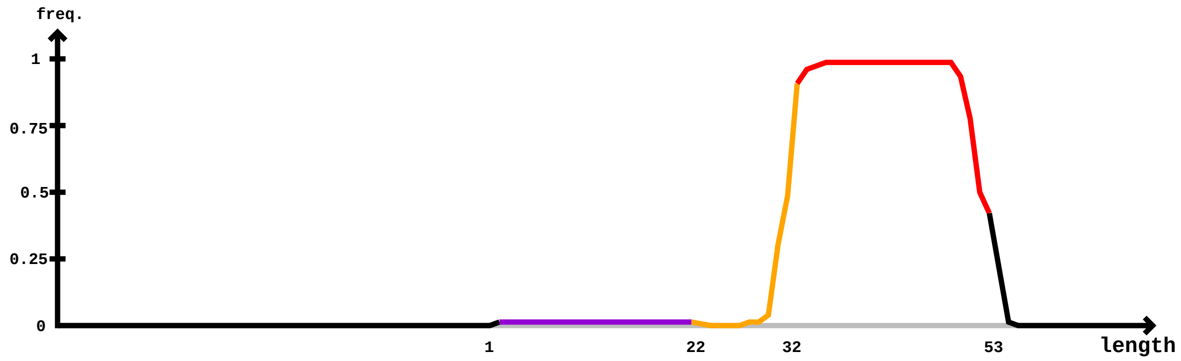


```
                                                  Star                          Mature
5'- uuaguccuugcagccacuuuaacaacagguccuuuggguuggccaaagaguucauuuggguuuuaccguacggaaaaacccgaaugaacuuuuuggcuaaucugauaccguc   -3'   obs
    uuaguccuugcagccacuuuaacaacagguccuuuggguuggccaaagaguucauuuggguuuuaccguacggaaaaacccgaaugaacuuuuuggcuaaucugauaccguc         exp
    ...........................(((...(((((((((((((((((((((((((((((((.((....)).))))))))))))))))))))))))))))))).)))...   reads   mm   sample
    ............................................aaagaguucauuuggguuuuC...............................................   1       1    seq
    ......................................................................cggaaaaacccgaaugaacuuu....................   1       0    seq
    ........................................................................gaaaaacccgaaugaacuuu....................   1       0    seq
    ........................................................................gaaaaaccAgaaugaacuuuu...................   1       1    seq
    .........................................................................aaaaaccAgaaugaacuuuu...................   10      1    seq
    .........................................................................aaaaacccgaaugaacuuuuG..................   10      1    seq
    ..........................................................................aaaaccAgaaugaacuuu....................   1       1    seq
    ..........................................................................aaaaccAgaaugaacuuuu...................   1       1    seq
    ..........................................................................aaaaccAgaaugaacuuuuu..................   7       1    seq
    ..........................................................................aaaacccUaaugaacuuuuu..................   1       1    seq
    ..........................................................................aaaacccgaaugaacuuuuG..................   2       1    seq
    ..........................................................................aaaaccGgaaugaacuuuuugg................   1       1    seq
    ..........................................................................aaaacccgaaugaacuuuuugg................   1       0    seq
    ...........................................................................aaacccgaaugaacuuu....................   1       0    seq
    ...........................................................................aaaccAgaaugaacuuuuug.................   1       1    seq
    ...........................................................................aaacccgaaugaaAuuuuug.................   1       1    seq
    ...........................................................................aaacccgaaugaacuuuuuC.................   1       1    seq
    ...........................................................................aaacccgaaugaacuuuuugg................   28      0    seq
    ............................................................................aacccgaaugaacuuuuu..................   1       0    seq
    ............................................................................aacccgaaugaacuuuuuC.................   1       1    seq
    ............................................................................aacccgaaugaacuuuuug.................   2       0    seq
    .............................................................................acccgaaugaacuuuuuggc...............   1       0    seq
    ..............................................................................cccgaaugaacuuuuuggcu..............   1       0    seq
```
